# Associations of eHealth literacy, physical literacy and physical fitness among urban middle school students in Guiyang, China: a multi-school cross-sectional study

**DOI:** 10.3389/fpubh.2026.1762497

**Published:** 2026-04-13

**Authors:** Junfeng Yuan, Lin Luo, Rui Wu

**Affiliations:** 1 School of Physical Education, Guizhou Normal University, Guiyang, China; 2 Key Laboratory of Brain Function and Brain Disease Prevention of Guizhou Province, Guiyang, China; 3 Guiyang No. 19 Middle School, Guiyang, China

**Keywords:** digital health education, eHealth literacy, health promotion, physical health, physical literacy, urban adolescents

## Abstract

**Objective:**

To assess the overall levels, disparities, and interrelations of eHealth literacy and physical literacy among urban junior high school students in Guiyang, examine their associations with physical health status, and develop a multifactorial predictive model to inform evidence-based health promotion strategies targeting urban adolescents.

**Methods:**

A stratified cluster sampling method was used to recruit students from grades 7 to 9 in multiple urban junior high schools across Guiyang. The study employed a cross-sectional quantitative design incorporating structured questionnaires and standardized physical fitness assessments. Validated instruments—including an established eHealth Literacy Scale and a physical literacy assessment tool—were used alongside data derived from the National Student Physical Fitness Standards. Demographic and familial educational background data were also collected. Descriptive statistics, non-parametric analyses, Spearman partial correlation, and multivariate logistic regression were conducted to systematically explore the relationships among eHealth literacy, physical literacy, and physical health levels.

**Results:**

Urban junior high school students in Guiyang demonstrated generally above-average levels of eHealth literacy, though a substantial subset fell below the expected proficiency threshold. Statistically meaningful disparities in both eHealth and physical literacy were observed across gender, grade level, parental education, and the frequency of digital health information instruction provided by families and teachers (*p* < 0.05). A robust positive association was identified between eHealth literacy and physical literacy (*r* = 0.412, *p* < 0.001), both of which were positively linked to higher physical health status. Multivariate regression analysis indicated that higher levels of “application ability” within eHealth literacy and stronger “motor skills” and “movement skills” within physical literacy were significantly associated with a lower likelihood of poor physical health status and a greater probability of belonging to higher physical health categories. Additionally, “health-related lifestyle awareness” had an independent effect on maintaining or improving qualified physical health levels.

**Conclusion:**

The physical health of urban junior high school students in Guiyang is shaped not only by their physical literacy but also by their capacity to navigate and utilize digital health information. Higher levels of eHealth literacy may facilitate adolescents’ ability to acquire and evaluate digital health information, which can further support the development of physical literacy and promote healthier behavioral patterns. Enhancing adolescent physical health therefore requires simultaneous investment in both digital health competencies and foundational physical literacy, supported by a coordinated framework across schools, families, and communities. This study elucidates the interactive dynamics among eHealth literacy, physical literacy, and physical health from an urban youth perspective, offering critical guidance for the design of localized digital health education, physical literacy enhancement, and integrated intervention strategies.

## 1 Introduction

In recent years, the decline in physical health among adolescents has emerged as a critical concern in China’s public health and education sectors. National surveillance data on student physical fitness indicate a fluctuating downward trend in core physical performance indicators—such as endurance, strength, and speed—among some junior high school students. Concurrently, the prevalence of health issues such as overweight/obesity and myopia continues to rise (1, 2). These physical health risks not only compromise adolescents’ daily learning efficiency and physical functioning (3) but may also exert long-term impacts on their adult health trajectories, chronic disease risks, and overall quality of life (4). As a key developmental window, adolescence is marked by rapid physical, psychological, and behavioral changes. Therefore, physical health in this stage not only reflects a student’s foundational capacity for holistic development but also serves as a vital indicator of the effectiveness of educational systems and national public health.

Health literacy has been recognized by the World Health Organization as a key determinant of health behaviors, decisions, and outcomes (5). For junior high school students, health literacy is crucial not only for acquiring, understanding, and applying health information but also for influencing participation in physical activity, lifestyle choices, and preventive health behaviors (6, 7). In the digital era, eHealth literacy—the ability to search for, evaluate, and apply health information from the internet and digital media to guide health behaviors—has become an essential component of adolescent health literacy (8). As “digital natives,” adolescents rely heavily on online information. However, insufficient eHealth literacy may expose them to misinformation or biased content, potentially leading to unhealthy lifestyles or misguided health decisions (9). From a theoretical perspective, adolescents with higher eHealth literacy may be better able to obtain accurate health information, critically evaluate digital content, and translate such information into healthier behavioral choices, including participation in physical activity and other health-promoting practices.

Simultaneously, physical literacy has emerged as a key concept in global physical and health education discourse. It encompasses a multidimensional set of attributes, including motor skills, physical competence, health-related knowledge, motivation, and confidence (10). Physical literacy is not only about the ability, willingness, and confidence to move but also forms the psychological and behavioral foundation for sustained engagement in physical activity and healthy living. Evidence indicates that higher physical literacy is significantly associated with greater physical activity levels, better athletic performance, and improved physical health outcomes (11, 12). Importantly, physical literacy may serve as a key behavioral and competence-based pathway linking health-related knowledge and information processing abilities to actual health outcomes. In this sense, adolescents who possess stronger eHealth literacy may be more capable of understanding health and exercise information, which in turn may facilitate the development of physical literacy and ultimately contribute to better physical health.

Existing research has primarily focused on the relationship between eHealth literacy and health behaviors among college students (13, 14), or the association between physical literacy and physical health in the same group (15), while insufficient attention has been paid to urban junior high school students during adolescence. In particular, limited empirical evidence exists regarding the interaction and pathways among eHealth literacy, physical literacy, and physical health in this demographic. Given the growing severity of adolescent health challenges, there is an urgent need to explore multifaceted determinants of adolescent physical health from an integrated perspective that includes both digital health literacy and physical literacy. Examining these constructs simultaneously may help clarify how digital health information competencies and movement-related capabilities jointly influence adolescent health outcomes.

This study aimed to assess the overall levels and disparities of eHealth literacy and physical literacy among urban junior high school students in Guiyang, explore their associations with physical health, and construct a multifactorial predictive model to inform locally grounded and actionable strategies for improving adolescent physical health. Based on the theoretical relationships among eHealth literacy, physical literacy, and physical health, the present study proposed the following hypotheses:

*H*1: eHealth literacy is positively associated with physical literacy among urban junior high school students.

*H*2: Both eHealth literacy and physical literacy are positively associated with adolescents’ physical health status.

*H*3: eHealth literacy and physical literacy jointly contribute to the prediction of adolescents’ physical health levels.

## 2 Subjects and methods

### 2.1 Study population

The study population comprised students from grades 7 to 9 in multiple urban junior high schools in Guiyang, Guizhou Province. Stratified cluster sampling was employed based on school region, school size, and grade distribution to ensure sample representativeness. Specifically, schools were selected from different urban districts of Guiyang to capture variability in school scale and educational environments, thereby improving the representativeness of the sample of urban junior high school students. All field investigators received standardized training prior to data collection to ensure consistency in questionnaire administration, response guidance, and data retrieval.

Before participation, the study’s objectives, content, and rights regarding informed consent were explained to students and their guardians. Written informed consent was obtained from both parties. Data collection was conducted in September 2023, yielding 1,757 valid questionnaires. The study adhered strictly to the ethical principles of the Declaration of Helsinki and was approved by the Academic Ethics Committee of Guizhou Normal University (Approval No.: 2023030005).

### 2.2 Instruments and variable measurement

#### 2.2.1 Demographic and background information

Collected data included student age, grade, self-rated health status, parental education level, and the frequency of digital health information education from teachers and family. These variables were treated as potential confounders in subsequent analyses.

#### 2.2.2 eHealth literacy

eHealth literacy was assessed using the eHealth Literacy Scale (eHEALS), originally developed by Norman (16) and adapted for the Chinese context by Guo et al. (17). The scale includes eight items rated on a 5-point Likert scale (1 = strongly disagree to 5 = strongly agree). Items 1–5 assess the ability to apply health information, items 6–7 evaluate information appraisal skills, and item 8 reflects health decision-making ability. Based on previous literature, total scores were categorized into low (8–16), moderate (17–31), and high (32–40) levels (11). The Cronbach’s *α* coefficient in this study was 0.969, indicating high internal consistency.

#### 2.2.3 Physical literacy

Physical literacy was measured using a Chinese version of the Physical Literacy Questionnaire developed by Luo et al. (18). The instrument includes 38 items across three domains and seven dimensions: motor skills (items 1–5), movement skills (6–14), physical activity behavior (15–16), physical activity cognition (17–20), health-related lifestyle awareness (21–24), confidence in participating in physical activity (25–26), and motivation to engage in physical activity (27–35). The Cronbach’s *α* coefficient in this study was 0.965, indicating good reliability.

#### 2.2.4 Physical health status

Physical health was assessed through on-site testing based on the National Student Physical Fitness Standards, which included BMI, vital capacity, 50-meter sprint, sit-and-reach test, one-minute sit-ups (for females) or pull-ups (for males). Overall scores were categorized as poor, qualified, good, or excellent.

### 2.3 Statistical methods

Data analysis was conducted using SPSS 23.0. The Shapiro–Wilk test was used to assess the normality of continuous variables. As eHealth literacy and physical literacy scores did not follow a normal distribution, non-parametric methods were applied: Mann–Whitney U test for two-group comparisons and Kruskal–Wallis H test for multiple-group comparisons. Results are presented as median (P_25_, P_75_). Spearman rank correlation analysis was used to explore associations, with covariates controlled as necessary. Variables included in the multivariate logistic regression model were selected based on theoretical relevance, prior literature, and the results of correlation analyses indicating potential associations with physical health outcomes. A multivariate logistic regression model was further used to examine the impact of eHealth literacy and physical literacy on physical health status. To ensure the stability of the regression model, multicollinearity among independent variables was assessed using variance inflation factors (VIF), and no serious multicollinearity was detected. All tests were two-sided with a significance level of *α* = 0.05.

## 3 Results

### 3.1 Differences in eHealth literacy among junior high school students by demographic characteristics

A total of 1,757 urban junior high school students in Guiyang were included in this study. The overall eHealth literacy scores ranged from 8 to 40, with a median score of 30.0 (P_25_ = 24.0, P_75_ = 32.0). Subscale scores were as follows: application ability ranged from 5 to 25 (median = 19.0, P_25_ = 15.0, P_75_ = 20.0), appraisal ability ranged from 2 to 10 (median = 7.0, P_25_ = 6.0, P_75_ = 8.0), and decision-making ability ranged from 1 to 5 (median = 4.0, P_25_ = 3.0, P_75_ = 4.0). According to previously established criteria, 3.81% of students demonstrated low eHealth literacy, 54.18% were at a moderate level, and 42.00% were at a high level, indicating an overall above-average proficiency, although a portion of students remained at risk of low eHealth literacy.

As shown in Table 1, significant differences in total and subscale eHealth literacy scores were observed by grade level, physical health status, frequency of digital health education from teachers, and frequency of digital health education from families (all *p* < 0.001). A clear upward trend in eHealth literacy was observed with increasing grade level and more frequent exposure to digital health education both at school and at home. Students’ decision-making ability scores differed significantly by fathers’ educational attainment (*p* < 0.05), and appraisal ability scores differed by mothers’ educational attainment (*p* < 0.05). However, no statistically significant differences in eHealth literacy scores were found based on age or gender (*p* > 0.05).

**Table 1:** Comparison of eHealth literacy scores and subscales by student characteristics.

Variable	n	Test statistic	Application ability	Appraisal ability	Decision-making ability	Total eHEALS score
Age						
11–12 years	214		19.000(15.0,20.0)	6.000(6.0,8.0)	3.000(3.0,4.0)	28.000(24.0,32.0)
13–14 years	1,311		20.000(15.0,20.0)	7.000(6.0,8.0)	4.000(3.0,4.0)	30.000(24.0,32.0)
15–16 years	232		20.000(15.3,20.0)	7.000(6.0,8.0)	4.000(3.0,4.0)	30.000(24.0,32.0)
		H statistic	3.475	4.805	4.867	4.096
		*p*-value	0.176	0.090	0.088	0.129
Gender						
Male	895		20.000(15.0,20.0)	7.000(6.0,8.0)	4.000(3.0,4.0)	30.000(24.0,32.0)
Female	862		19.500(15.0,20.0)	7.000(6.0,8.0)	4.000(3.0,4.0)	30.000(24.0,32.0)
		U statistic	381,836	383050.5	381,189	382,567
		z statistic	−0.375	−0.262	−0.453	−0.301
		*p*-value	0.708	0.793	0.650	0.763
Grade						
Grade 7	810		19.000(15.0,20.0)	6.000(6.0,8.0)	3.000(3.0,4.0)	29.000(24.0,32.0)
Grade 8	666		20.000(16.0,20.0)	8.000(6.0,8.0)	4.000(3.0,4.0)	31.000(25.0,32.0)
Grade 9	281		20.000(15.5,20.0)	7.000(6.0,8.0)	4.000(3.0,4.0)	30.000(24.0,32.0)
		H statistic	23.904	24.391	16.480	23.938
		*p*-value	<0.001	<0.001	<0.001	<0.001
Father’s education level						
Junior high school or below	363		19.000(15.0,20.0)	7.000(6.0,8.0)	4.000(3.0,4.0)	30.000(24.0,32.0)
Senior high school / Technical secondary school / Vocational college	371		20.000(15.0,20.0)	8.000(6.0,8.0)	4.000(3.0,4.0)	31.000(25.0,32.0)
Junior college / Bachelor’s degree	885		20.000(15.0,20.0)	7.000(6.0,8.0)	4.000(3.0,4.0)	30.000(24.0,32.0)
Postgraduate	138		20.000(15.0,20.0)	7.500(6.0,8.0)	4.000(3.0,4.0)	30.500(24.0,32.0)
		H statistic	1.419	5.731	8.004	3.800
		*p*-value	0.701	0.125	0.046	0.284
Mother’s education level						
Junior high school or below	405		20.000(15.5,20.0)	7.000(6.0,8.0)	4.000(3.0,4.0)	30.000(24.0,32.0)
Senior high school / Technical secondary school / Vocational college	366		20.000(16.0,20.0)	7.500(6.0,8.0)	4.000(3.0,4.0)	30.000(25.0,32.0)
Junior college / Bachelor’s degree	874		19.000(15.0,20.0)	7.000(6.0,8.0)	4.000(3.0,4.0)	29.000(24.0,32.0)
Postgraduate	112		20.000(15.0,20.0)	8.000(6.0,8.0)	4.000(3.0,4.0)	31.000(24.0,32.0)
		H statistic	4.922	9.081	7.293	6.151
		*p*-value	0.178	0.028	0.063	0.104
Frequency of digital health information education by teachers						
Rarely	338		15.000(14.0,20.0)	6.000(5.0,7.0)	3.000(3.0,4.0)	24.000(22.0,30.0)
Sometimes	920		19.000(15.0,20.0)	6.000(6.0,8.0)	3.000(3.0,4.0)	28.000(24.0,32.0)
Often	449		20.000(20.0,25.0)	8.000(8.0,10.0)	4.000(4.0,5.0)	32.000(32.0,40.0)
		H statistic	400.677	425.876	390.195	443.667
		*p*-value	<0.001	<0.001	<0.001	<0.001
Frequency of digital health information education by family						
Rarely	461		15.000(15.0,19.0)	6.000(5.0,7.0)	3.000(3.0,4.0)	24.000(22.0,29.0)
Sometimes	882		19.000(15.0,20.0)	7.000(6.0,8.0)	4.000(3.0,4.0)	29.000(25.0,32.0)
Often	464		20.000(20.0,25.0)	8.000(8.0,10.0)	4.000(4.0,5.0)	32.000(32.0,40.0)
		H statistic	475.408	454.527	429.773	507.801
		*p*-value	<0.001	<0.001	<0.001	<0.001
Physical health status						
Poor	88		19.000(15.0,20.0)	6.000(6.0,8.0)	3.000(3.0,4.0)	28.000(24.0,32.0)
Qualified	621		19.000(15.0,20.0)	6.000(6.0,8.0)	3.000(3.0,4.0)	29.000(24.0,32.0)
Good	572		19.000(15.0,20.0)	7.000(6.0,8.0)	3.000(3.0,4.0)	29.000(24.0,32.0)
Excellent	476		20.000(17.0,25.0)	8.000(6.0,10.0)	4.000(3.0,5.0)	32.000(26.0,39.0)
		H statistic	61.699	62.230	65.644	67.245
		*p*-value	<0.001	<0.001	<0.001	<0.001

Data presented as median (P25, P75).

### 3.2 Differences in physical literacy among junior high school students by demographic characteristics

The physical literacy scores of junior high school students were as follows: motor skills ranged from 6 to 25 points (mean = 20.72 ± 3.32), movement skills from 9 to 45 (mean = 32.69 ± 6.91), physical activity from 2 to 10 (mean = 7.59 ± 1.84), physical activity cognition from 4 to 20 (mean = 16.24 ± 2.79), health-related lifestyle awareness from 4 to 20 (mean = 15.35 ± 3.21), confidence in physical activity from 3 to 15 (mean = 10.45 ± 2.59), and motivation for physical activity from 11 to 55 (mean = 40.17 ± 8.08).

As shown in Table 2, students of different ages showed significant differences in movement skill scores (*p* < 0.05), with a slight decreasing trend among older students. Gender differences were mainly observed in the physical activity dimension, where boys scored significantly higher than girls (*p* < 0.01), indicating that boys may engage in physical activities more frequently or intensely.

**Table 2:** Comparison of physical literacy scores and subscales by student characteristics.

Variable	n	Test statistic	Motor skills	Movement skills	Physical activity	Physical-activity cognition	Health-related lifestyle awareness	Confidence in physical activity	Motivation for physical activity	Total physical literacy score
Age										
11–12 years	214		21.000(19.0,24.0)	34.000(29.0,37.0)	8.000(7.0,9.0)	16.000(15.0,19.0)	16.000(13.8,18.0)	10.000(9.0,12.0)	39.000(34.0,44.0)	143.000(131.0,157.0)
13–14 years	1,311		21.000(19.0,24.0)	33.000(28.0,37.0)	8.000(6.0,9.0)	16.000(15.0,18.0)	16.000(13.0,18.0)	10.000(9.0,12.0)	41.000(34.0,44.0)	143.000(127.0,157.0)
15–16 years	232		20.000(18.0,23.0)	32.000(27.0,37.0)	8.000(6.0,9.0)	16.000(14.3,18.0)	16.000(13.0,18.0)	11.000(9.0,12.0)	41.000(35.0,44.0)	141.000(127.0,156.8)
		H statistic	5.077	6.432	1.185	5.455	0.489	1.255	2.000	1.493
		*p*-value	0.079	0.040	0.553	0.065	0.783	0.534	0.368	0.474
Gender										
Male	895		21.000(19.0,24.0)	33.000(28.0,37.0)	8.000(7.0,9.0)	16.000(15.0,19.0)	16.000(13.0,18.0)	10.000(9.0,12.0)	41.000(34.0,44.0)	143.000(129.0,157.0)
Female	862		21.000(19.0,23.0)	33.000(28.0,37.0)	8.000(6.0,9.0)	16.000(15.0,18.0)	16.000(13.0,18.0)	11.000(9.0,12.0)	40.000(34.0,44.0)	143.000(127.0,157.0)
		U statistic	376,955	368110.5	351835.5	382121.5	381,402	375800.5	379566.5	374256.5
		z statistic	−0.832	−1.661	−3.243	−0.345	−0.413	−0.948	−0.583	−1.081
		*p*-value	0.406	0.097	0.001	0.730	0.679	0.343	0.56	0.28
Grade										
Grade 7	810		21.000(19.0,23.0)	33.000(28.0,37.0)	8.000(6.0,9.0)	16.000(15.0,18.0)	16.000(13.0,17.0)	10.000(9.0,12.0)	39.000(34.0,44.0)	141.000(127.0,155.0)
Grade 8	666		21.000(19.0,24.0)	34.000(29.0,38.0)	8.000(6.0,9.0)	16.000(15.0,19.0)	16.000(14.0,18.0)	11.000(9.0,12.0)	42.000(35.0,45.0)	147.000(131.0,159.0)
Grade 9	281		20.000(18.0,23.0)	32.000(27.0,36.5)	7.000(6.0,9.0)	16.000(14.0,18.0)	16.000(13.0,18.0)	11.000(9.0,12.0)	41.000(34.0,44.0)	140.000(126.5,157.0)
		H statistic	11.831	15.543	8.454	1.590	9.956	19.108	16.130	17.185
		*p*-value	0.003	<0.001	0.015	0.452	0.007	<0.001	<0.001	<0.001
Father’s education level										
Junior high school or below	363		20.000(18.0,23.0)	32.000(27.0,36.0)	8.000(6.0,9.0)	16.000(15.0,19.0)	16.000(14.0,18.0)	10.000(9.0,12.0)	40.000(34.0,44.0)	140.000(126.0,156.0)
Senior high school / Technical secondary school / Vocational college	371		21.000(19.0,24.0)	34.000(28.0,38.0)	8.000(6.0,9.0)	16.000(15.0,19.0)	16.000(14.0,18.0)	11.000(9.0,12.0)	41.000(34.0,45.0)	146.000(130.0,161.0)
Junior college / Bachelor’s degree	885		21.000(19.0,23.0)	33.000(28.0,37.0)	8.000(6.0,9.0)	16.000(15.0,18.0)	16.000(13.0,17.0)	10.000(9.0,12.0)	40.000(34.0,44.0)	143.000(129.0,156.0)
Postgraduate	138		21.000(18.8,24.0)	34.000(28.0,37.3)	8.000(6.0,9.0)	16.000(15.0,18.0)	15.000(12.0,16.0)	11.000(9.0,12.0)	41.000(34.0,44.0)	143.000(127.8,155.3)
		H statistic	14.402	12.749	3.987	4.620	16.300	2.311	2.801	7.522
		*p*-value	0.002	0.005	0.263	0.202	0.001	0.510	0.423	0.057
Mother’s education level										
Junior high school or below	405		20.000(18.0,23.0)	32.000(27.0,36.0)	8.000(6.0,9.0)	16.000(15.0,19.0)	16.000(14.0,18.0)	10.000(9.0,12.0)	41.000(35.0,44.0)	141.000(127.0,157.5)
Senior high school / Technical secondary school / Vocational college	366		21.000(19.0,24.0)	33.000(28.0,37.3)	8.000(6.0,9.0)	16.000(15.0,19.0)	16.000(14.0,18.0)	11.000(9.0,12.0)	43.000(35.0,45.0)	146.000(130.0,159.3)
Junior college / Bachelor’s degree	874		21.000(19.0,24.0)	34.000(28.0,37.0)	8.000(6.0,9.0)	16.000(15.0,18.0)	16.000(13.0,17.0)	10.000(9.0,12.0)	39.000(34.0,44.0)	143.000(128.0,156.0)
Postgraduate	112		21.000(19.0,24.0)	34.000(29.0,39.0)	8.000(7.0,9.0)	16.000(15.0,18.0)	15.000(12.0,16.8)	11.000(9.0,12.0)	41.000(34.0,44.0)	145.500(126.0,158.5)
		H statistic	12.164	7.445	3.456	2.499	14.285	3.143	11.468	3.842
		*p*-value	0.007	0.059	0.326	0.475	0.003	0.370	0.009	0.279
Frequency of digital health information education by teachers										
Rarely	338		20.000(19.0,23.0)	31.000(26.0,36.0)	8.000(6.0,9.0)	16.000(14.0,18.0)	14.000(12.0,16.0)	9.000(7.0,10.0)	34.500(31.0,41.0)	134.000(117.8,146.0)
Sometimes	920		20.000(18.0,23.0)	32.500(28.0,36.0)	8.000(6.0,9.0)	16.000(14.0,17.0)	16.000(13.0,16.0)	10.000(9.0,12.0)	39.000(34.0,44.0)	139.000(126.0,151.0)
Often	449		22.000(20.0,25.0)	36.000(30.0,41.0)	8.000(7.0,10.0)	18.000(16.0,20.0)	17.000(16.0,20.0)	12.000(11.0,15.0)	44.000(42.0,55.0)	158.000(144.0,176.0)
		H statistic	96.227	96.326	96.616	160.152	220.54	420.98	392.848	314.186
		*p*-value	<0.001	<0.001	<0.001	<0.001	<0.001	<0.001	<0.001	<0.001
Frequency of digital health information education by family										
Rarely	461		20.000(18.0,23.0)	31.000(26.0,36.0)	7.000(6.0,8.0)	16.000(14.0,17.0)	14.000(12.0,16.0)	9.000(7.0,10.0)	34.000(31.0,40.5)	131.000(118.0,144.0)
Sometimes	882		21.000(18.0,23.0)	33.000(28.0,36.0)	8.000(6.0,9.0)	16.000(15.0,18.0)	16.000(13.0,16.0)	10.000(9.0,12.0)	39.000(35.0,44.0)	141.000(127.0,153.0)
Often	464		23.000(20.0,25.0)	36.000(32.0,41.0)	9.000(8.0,10.0)	18.000(16.0,20.0)	18.000(16.0,20.0)	12.000(11.0,15.0)	45.000(43.0,55.0)	161.000(147.0,177.0)
		H statistic	145.956	134.438	142.173	212.434	274.386	466.708	497.118	415.958
		*p*-value	<0.001	<0.001	<0.001	<0.001	<0.001	<0.001	<0.001	<0.001
Physical health status										
Poor	88		17.500(14.3,21.0)	24.000(21.0,31.5)	6.000(5.0,8.0)	15.000(12.0,16.8)	12.000(11.0,16.0)	10.000(8.0,12.0)	39.000(33.0,44.0)	121.000(108.3,142.5)
Qualified	621		20.000(18.0,22.0)	30.000(26.0,35.0)	7.000(6.0,8.0)	16.000(14.0,17.0)	16.000(12.0,16.0)	10.000(9.0,12.0)	39.000(34.0,44.0)	136.000(123.0,150.0)
Good	572		21.000(19.0,23.0)	33.000(28.0,36.0)	8.000(6.0,9.0)	16.000(15.0,18.0)	16.000(13.0,17.0)	10.000(9.0,12.0)	40.000(34.0,44.0)	142.000(129.0,154.8)
Excellent	476		23.000(21.0,25.0)	37.000(33.0,42.0)	9.000(8.0,10.0)	18.000(16.0,20.0)	16.000(15.0,20.0)	12.000(9.0,13.0)	43.000(36.0,51.0)	156.000(143.0,173.0)
		H statistic	289.466	334.256	245.218	165.515	123.98	53.538	69.976	271.048
		*p*-value	<0.001	<0.001	<0.001	<0.001	<0.001	<0.001	<0.001	<0.001

Data presented as median (P25, P75).

Grade-level differences were notable: motor skills, movement skills, physical activity, health-related lifestyle awareness, confidence, motivation, and overall physical literacy scores all differed significantly across grades (*p* < 0.05), with Grade 8 students generally achieving higher scores than those in Grade 7 and Grade 9.

Regarding socio-cultural family background, students with fathers of different educational levels showed significant differences in motor skills, movement skills, and health-related lifestyle awareness (*p* < 0.01). Similarly, students with mothers of different educational levels had significant differences in motor skills, health-related lifestyle awareness, and motivation for physical activity (*p* < 0.05), suggesting that parental education may influence students’ physical literacy through health beliefs and role modeling.

Support from teachers and families in health education was strongly associated with physical literacy. Significant differences in all dimensions and total physical literacy scores were observed based on the frequency of digital health education provided by teachers and families (all *p* < 0.001). A positive trend was evident, with higher frequency of education linked to higher scores in motor and movement skills, physical activity, health-related lifestyle awareness, confidence, motivation, and total physical literacy.

Physical health status was also significantly associated with all dimensions and the total score of physical literacy (all *p* < 0.001). Compared to the “poor” group, students in the “qualified,” “good,” and “excellent” groups showed progressively higher scores across motor and movement skills, physical activity, health-related lifestyle awareness, motivation, and total physical literacy—demonstrating a clear gradient: better physical health was linked with higher physical literacy.

### 3.3 Associations among eHealth literacy, physical literacy, and physical health

Partial correlation analyses were conducted while controlling for gender, age, parental education, and the frequency of digital health information education by teachers and families (Tables 3, 4). The results showed that grade level was significantly positively correlated with all eHealth literacy dimensions and total score (*r* = 0.080–0.097, *p* < 0.01). The frequency of digital health education by teachers (*r* = 0.454–0.483, *p* < 0.01) and families (*r* = 0.483–0.528, *p* < 0.01) demonstrated moderate positive correlations with eHealth literacy, indicating that greater emphasis on digital health education at school and home was associated with higher levels of eHealth literacy among students.

**Table 3:** Correlation analysis between variables and control factors.

Variables	Gender	Age	Grade	Father’s education level	Mother’s education level	Frequency of digital health education by teachers	Frequency of digital health education by family
Application ability	−0.004	0.018	0.089**	0	−0.038	0.456**	0.511**
Appraisal ability	−0.005	0.022	0.097**	−0.004	−0.043	0.476**	0.500**
Decision-making ability	−0.011	0.022	0.080**	−0.008	−0.032	0.454**	0.483**
eHEALS total score	−0.005	0.02	0.094**	−0.002	−0.04	0.483**	0.528**
Motor skills	−0.015	−0.045	−0.001	0.074**	0.074**	0.175**	0.248**
Movement skills	−0.03	−0.062**	−0.012	0.064**	0.055*	0.216**	0.263**
Physical activity	−0.077**	−0.032	−0.028	−0.026	−0.032	0.186**	0.235**
Cognition of physical activity	0.005	−0.055*	0	−0.006	−0.028	0.228**	0.309**
Health-related lifestyle awareness	−0.009	−0.01	0.053*	−0.074**	−0.082**	0.331**	0.374**
Confidence in physical activity	0.025	0.03	0.086**	0.007	−0.007	0.483**	0.508**
Motivation for physical activity	−0.013	0.021	0.072**	−0.005	−0.039	0.465**	0.524**
Total physical literacy score	−0.02	−0.026	0.037	0.016	−0.005	0.410**	0.481**
Physical health level	−0.002	−0.001	0.013	0.066**	0.068**	0.078**	0.096**

*p < 0.05; **p < 0.01.

**Table 4:** Partial correlation analysis between eHealth literacy, physical literacy, and physical health status.

Variables	1	2	3	4	5	6	7	8	9	10	11	12
1. Application ability	1											
2. Appraisal ability	0.781**	1										
3. Decision-making ability	0.755**	0.810**	1									
4. eHEALS total score	0.970**	0.900**	0.858**	1								
5. Motor skills	0.268**	0.269**	0.260**	0.285**	1							
6. Movement skills	0.219**	0.251**	0.267**	0.250**	0.703**	1						
7. Physical activity	0.276**	0.247**	0.264**	0.285**	0.538**	0.593**	1					
8. Cognition of physical activity	0.344**	0.301**	0.297**	0.349**	0.487**	0.490**	0.551**	1				
9. Health-related lifestyle awareness	0.354**	0.344**	0.325**	0.371**	0.517**	0.551**	0.552**	0.678**	1			
10. Confidence in physical activity	0.600**	0.579**	0.559**	0.629**	0.236**	0.295**	0.278**	0.268**	0.349**	1		
11. Motivation for physical activity	0.751**	0.719**	0.707**	0.787**	0.303**	0.312**	0.301**	0.354**	0.411**	0.712**	1	
12. Total physical literacy score	0.577**	0.565**	0.560**	0.610**	0.737**	0.810**	0.683**	0.696**	0.756**	0.618**	0.727**	1
13. Physical health level	0.117**	0.133**	0.129**	0.131**	0.384**	0.424**	0.356**	0.279**	0.252**	0.127**	0.163**	0.391**

*p < 0.05; **p < 0.01.

Regarding physical literacy, parental education level showed weak but significant positive correlations with some physical literacy dimensions (father: *r* = 0.064–0.074, *p* < 0.01; mother: *r* = 0.055–0.074, *p* < 0.05). The frequency of digital health education from teachers and families was moderately to strongly correlated with physical literacy dimensions and total scores (*r* = 0.175–0.524, *p* < 0.01), suggesting that digital health education may not only enhance eHealth literacy but also indirectly improve physical literacy by promoting health knowledge and behavior.

Physical health status was also significantly positively correlated with parental education and both sources of digital health education (all *p* < 0.01).

Further partial correlation analyses (Table 4) revealed significant positive correlations between all dimensions and the total scores of eHealth literacy and physical literacy (*r* = 0.219–0.371, *p* < 0.01). This indicates that higher eHealth literacy is associated with better motor skills, movement skills, physical activity, health-related lifestyle awareness, confidence, and motivation.

eHealth literacy was also positively correlated with physical health status (*r* = 0.117–0.133, *p* < 0.01), albeit at a weaker level, suggesting that improving eHealth literacy may contribute to better physical health.

Correlations between physical literacy and physical health status were stronger (*r* = 0.236–0.424, *p* < 0.01), supporting the critical role of physical literacy in shaping physical health outcomes—students with higher motor and movement skills, and stronger health-related awareness, tended to exhibit better physical health.

### 3.4 Logistic regression analysis of factors influencing physical health status in junior high school students

A multinomial logistic regression model was constructed using “poor” physical health status as the reference group. Independent variables included the subscales of eHealth literacy, physical literacy, and the frequency of digital health education from families. The results are presented in Table 5.

**Table 5:** Logistic regression analysis of factors influencing physical health status among junior high school students.

Variable	Qualified		Good		Excellent	
	*β (p)*	*OR (95% CI)*	*β (p)*	*OR (95% CI)*	*β (p)*	*OR (95% CI)*
Application ability	0.162 (0.006)	0.85 (0.757–0.955)	0.121 (0.045)	0.886 (0.787–0.997)	0.172 (0.009)	0.842 (0.740–0.958)
Appraisal ability	0.183 (0.157)	1.20 (0.932–1.546)	0.139 (0.294)	1.149 (0.886–1.489)	0.227 (0.120)	1.255 (0.943–1.670)
Decision-making ability	0.355 (0.167)	0.701 (0.424–1.160)	0.479 (0.068)	0.619 (0.370–1.036)	0.275 (0.338)	0.760 (0.433–1.332)
Motor skills	0.102 (0.030)	1.107 (1.010–1.214)	0.123 (0.011)	1.131 (1.029–1.244)	0.242 (<0.001)	1.273 (1.145–1.415)
Movement skills	0.056 (0.038)	1.057 (1.003–1.114)	0.100 (<0.001)	1.106 (1.048–1.167)	0.167 (<0.001)	1.182 (1.116–1.251)
Physical activity	0.040 (0.603)	1.041 (0.896–1.209)	0.139 (0.079)	1.149 (0.984–1.341)	0.312 (<0.001)	1.366 (1.150–1.623)
Health-related lifestyle awareness	0.126 (0.023)	1.134 (1.017–1.264)	0.082 (0.146)	1.086 (0.972–1.213)	0.016 (0.789)	0.984 (0.874–1.108)
Family digital health education freq	0.518 (0.019)	0.596 (0.386–0.920)	0.469 (0.038)	0.626 (0.402–0.974)	0.568 (0.017)	0.567 (0.355–0.905)

The regression analysis revealed that, after controlling for other variables, “application ability” in eHealth literacy emerged as a significant predictor across all three physical health categories—“qualified,” “good,” and “excellent” (*p* < 0.05). This suggests that higher levels of health information application ability are associated with a lower likelihood of remaining in the “poor” physical health group and a greater probability of belonging to higher physical health categories.

In terms of physical literacy, both “motor skills” and “movement skills” were significant predictors across all three categories (*p* < 0.05), indicating that stronger foundational movement competence is associated with a reduced risk of poor physical health and with increased odds of achieving higher physical health levels.

Moreover, the “physical activity” dimension showed a significant effect in the “excellent” category (*p* < 0.01), suggesting that greater engagement in physical activity is associated with higher odds of reaching the excellent physical health category.

“Health-related lifestyle awareness” was a significant predictor in the “qualified” group (*p* < 0.05), highlighting the importance of health knowledge and lifestyle cognition in helping students move from poor to at least qualified physical health status.

Family digital health education frequency was a significant influencing factor across all three higher physical health groups (*p* < 0.05), indicating that parental involvement in providing digital health information and guiding healthy behavior is associated with improved physical health status among adolescents.

Taken together, application ability in eHealth literacy, motor skills and movement skills in physical literacy, and family digital health education frequency emerged as key factors associated with higher physical health levels. Meanwhile, physical activity and lifestyle awareness provide additional support in achieving and maintaining higher physical health.

## 4 Discussion

This study revealed that urban junior high school students in Guiyang demonstrated an overall above-average level of eHealth literacy, although a notable proportion still exhibited low literacy levels. On one hand, this may be attributed to the region’s relatively advanced information infrastructure and the progressive implementation of health and information technology education in schools. On the other hand, it also highlights that although adolescents are often regarded as “digital natives,” their eHealth literacy does not automatically improve with increased internet use, and still requires structured educational interventions (19, 20). The significant upward trend of eHealth literacy across grade levels may reflect the gradual enhancement of abstract thinking, critical thinking, and academic autonomy during adolescence, which in turn improves students’ ability to filter, comprehend, and synthesize health information (21, 22). International research similarly supports the notion that early and continuous digital health education helps adolescents develop stable health information processing patterns, ultimately shaping long-term health behaviors and outcomes (21, 22).

This study also found a moderate positive correlation between the frequency of digital health education provided by teachers and students’ eHealth literacy, validating the “education supply—capability enhancement—behavioral change” mechanism. As a formal educational setting, schools can effectively strengthen students’ judgment of online health information credibility and foster sound usage habits by integrating digital health education into curricula, classroom discussions, and extracurricular activities. Family background factors also play a significant role in shaping eHealth literacy. Households with more educated parents often provide richer health information environments and more proactive health values, which can subtly enhance adolescents’ abilities to process health information (21, 23). Our findings show that the frequency of digital health education within the family is a strong predictor of eHealth literacy, suggesting that families are not only providers of health information but also influence adolescents’ health decision-making styles and the development of critical thinking (24). Compared to international experiences, China still faces challenges in building a comprehensive mechanism for school-family collaboration in digital health education. Future strategies should include curriculum reform, parental training, and community resource integration to establish a cooperative “school-family” framework for digital health education.

Regarding physical literacy, the study found that male students performed better in motor skills and physical activity dimensions, while females scored higher in health-related lifestyle awareness. These differences may relate to gender socialization, extracurricular activity preferences, and differences in school physical education programming (25). Grade-level differences revealed that most physical literacy dimensions improved with advancing grades, reflecting the cumulative effects of physiological development, physical education exposure, and learning experiences (26). However, third-year students showed slight declines in some areas, likely due to increased academic pressure limiting physical activity—aligning with the risk pathway of “academic stress—reduced exercise—compromised physical health.” In the context of China’s examination-oriented education system, students in higher grades often face increasing academic workloads and reduced discretionary time, which may constrain opportunities for regular physical activity and partially explain the observed decline in certain dimensions of physical literacy.

Socio-cultural family background also significantly impacted physical literacy. Fathers’ educational levels were primarily associated with students’ motor and movement skills as well as lifestyle awareness, while mothers’ education was linked to motor skills, lifestyle cognition, and physical activity motivation, suggesting differentiated roles within family health behavior formation. Furthermore, the frequency of digital health education from both teachers and families was significantly associated with all dimensions of physical literacy. This indicates that digital health education may not only enhance information processing but also foster improvements in health knowledge, self-efficacy, and health responsibility, thereby promoting participation in physical activity and development of motor skills (27, 28). The moderate positive correlation between physical literacy and physical health status further underscores the key role of physical literacy as a “critical nexus” in supporting lifelong physical activity and improving physical health outcomes (27, 28).

This study also identified a significant positive correlation between eHealth literacy and physical literacy, providing empirical support for the theoretical chain of “information literacy—behavioral capacity—health outcomes.” Students with higher eHealth literacy are better equipped to identify high-quality health and exercise information, thereby adopting more scientific training methods and healthier lifestyles. They also exhibit greater risk awareness regarding sedentary behavior and obesity, prompting them to proactively adjust physical activity levels and lifestyle habits. Although the correlation between eHealth literacy and physical health was slightly weaker than that between physical literacy and physical health, it remained significantly positive—supporting the “information—motivation—behavior—outcome” model proposed in health literacy frameworks (29). The stronger correlation between physical literacy and physical health reflects a more direct mechanism of action: “movement ability—participation—health improvement.” These findings suggest that physical literacy may function as a potential behavioral pathway through which eHealth literacy influences physical health outcomes. In other words, adolescents with stronger digital health information competencies may be more capable of translating health knowledge into movement-related behaviors, thereby enhancing their physical literacy and ultimately improving physical health.

Together, these findings suggest that eHealth literacy and physical literacy play a synergistic role in promoting adolescent physical health. The former provides the informational and cognitive foundation, while the latter supplies the skills and behavioral foundation. This synergistic relationship highlights the importance of integrating digital health education with physical education programs, allowing adolescents to simultaneously develop health information competencies and movement-related capabilities. Future research could employ longitudinal designs and structural equation modeling to further verify this synergy and explore potential mediating and moderating pathways.

The multinomial logistic regression analysis confirmed the predictive roles of both eHealth literacy and physical literacy in determining physical health status. Specifically, “application ability” in eHealth literacy and “motor skills” and “movement skills” in physical literacy were key predictors associated with higher physical health categories, while “health-related lifestyle awareness” was crucial in differentiating between “qualified” and “poor” groups. These findings suggest the need for a tiered and categorized intervention strategy: for students with lower physical health, emphasis should be placed on improving basic motor skills and health cognition to help them meet minimum standards. For those already meeting or exceeding basic health criteria, interventions should focus on specialized physical capacity and skill development to further advance health status. Additionally, the frequency of family-provided digital health education remained a significant factor across all physical health categories, highlighting the long-term and consistent role families play in shaping daily exercise habits, dietary behaviors, and health information engagement.

From a public health and school physical education perspective, the findings offer several practical implications for youth health promotion. Schools should integrate eHealth literacy education into health and physical education curricula through case-based learning, media literacy training, and project-based approaches. Physical education should systematically incorporate the concept of physical literacy, shifting instructional goals from merely improving fitness to fostering the integrated capability of “being able, willing, and understanding movement.” For example, schools may introduce modules that teach students how to evaluate online health information, combine digital health learning with practical exercise guidance, and design school-based physical activity programs supported by digital health platforms. At the family level, parental training and health-related task guidance can enhance the quality of home-based digital health education, forming a supportive health environment. Communities and societal organizations can contribute by providing open access to physical activity facilities, developing digital health platforms, and conducting health promotion campaigns. Ultimately, a comprehensive intervention framework involving coordinated efforts from schools, families, and communities should be established to simultaneously enhance eHealth literacy, physical literacy, and physical health outcomes.

This study has several strengths. First, it is among the first to systematically integrate eHealth literacy, physical literacy, and physical health into a unified framework for urban junior high school students, offering a comprehensive perspective on adolescent health literacy, behavior, and outcomes. Second, the use of multi-school cluster sampling ensures a large and representative sample, enabling reliable assessment of the urban junior high school population in Guiyang. Third, incorporating both teacher and family digital health education into the analysis helps elucidate the broader socio-ecological mechanisms underlying the development of eHealth and physical literacy. Fourth, the use of partial correlation and multinomial logistic regression enables the construction of a predictive model for physical health, enhancing the applicability and practical relevance of the findings.

Nevertheless, this study has some limitations. First, its cross-sectional design precludes causal inference, and the directionality of associations among eHealth literacy, physical literacy, and physical health requires longitudinal validation. Second, since the sample was drawn exclusively from urban schools, generalizability to rural or other populations may be limited. Third, some indicators relied on self-reporting, which could be subject to recall bias and social desirability effects. Lastly, this study did not apply structural equation modeling to explore mediation or moderation pathways between eHealth and physical literacy, which future research should address by expanding methodological approaches. Despite these limitations, the study offers important theoretical support and context-specific evidence for youth health promotion.

## 5 Conclusion

This study investigated the relationships among eHealth literacy, physical literacy, and physical health status in urban junior high school students in Guiyang. Results showed that although students overall exhibited above-average eHealth literacy, a non-negligible proportion still had low levels. Significant disparities were found across grade levels, family education background, and the frequency of digital health education at both school and home. A significant positive correlation was observed between eHealth literacy and physical literacy, and both were positively associated with physical health status. Among them, the “application ability” dimension of eHealth literacy and the “motor skills” and “movement skills” of physical literacy were important predictors associated with higher physical health levels. “Health-related lifestyle awareness” played a key role in distinguishing between “qualified” and “poor” health groups. These findings highlight the interconnected roles of digital health information competencies and movement-related capabilities in shaping adolescent physical health.

These findings suggest that improving adolescent physical health cannot rely solely on traditional physical exercise. It is essential to incorporate digital health information skills and physical literacy cultivation into comprehensive health promotion strategies. In particular, schools should strengthen digital health education and integrate it with physical education curricula, families should actively guide adolescents in accessing and evaluating online health information, and communities should provide supportive environments and resources that encourage active lifestyles. By building an integrated intervention system centered on schools, supported by families, and extended through communities, we can systematically strengthen both health information literacy and physical literacy. Such efforts will help achieve synergistic improvements in eHealth literacy, physical literacy, and physical health, thereby laying a solid foundation for healthier developmental trajectories in adolescence and chronic disease prevention in adulthood.

## Data Availability

The raw data supporting the conclusions of this article will be made available by the authors, without undue reservation.
